# Dynamic whole-body [^18^F]FES PET/CT increases lesion visibility in patients with metastatic breast cancer

**DOI:** 10.1186/s13550-024-01080-y

**Published:** 2024-03-04

**Authors:** Mette A. Pedersen, Ole L. Munk, André H. Dias, Johanne H. Steffensen, Anders L. Møller, Anna Lyhne Johnsson, Kim Vang Hansen, Dirk Bender, Steen Jakobsen, Morten Busk, Lars C. Gormsen, Trine Tramm, Signe Borgquist, Mikkel H. Vendelbo

**Affiliations:** 1 Department of Nuclear Medicine & PET-Centre, Aarhus University Hospital, Palle-Juul-Jensens Boulevard 165, 8200 Aarhus, Denmark; 2 Department of Biomedicine, Aarhus University, Aarhus, Denmark; 3 Steno Diabetes Center Aarhus, Aarhus University Hospital, Aarhus, Denmark; 4 Department of Clinical Medicine, Aarhus University, Aarhus, Denmark; 5 Department of Oncology, Aarhus University Hospital, Aarhus, Denmark; 6 Department of Radiology, Aarhus University Hospital, Aarhus, Denmark; 7 Department of Experimental Clinical Oncology, Aarhus University Hospital, Aarhus, Denmark; 8 Danish Centre for Particle Therapy, Aarhus University Hospital, Aarhus, Denmark; 9 Department of Pathology, Aarhus University Hospital, Aarhus, Denmark

**Keywords:** [^18^F]FES, Dynamic whole-body PET/CT, Tumor visibility, Breast cancer, Estrogen receptor

## Abstract

**Background:**

Correct classification of estrogen receptor (ER) status is essential for prognosis and treatment planning in patients with breast cancer (BC). Therefore, it is recommended to sample tumor tissue from an accessible metastasis. However, ER expression can show intra- and intertumoral heterogeneity. 16α-[^18^F]fluoroestradiol ([^18^F]FES) Positron Emission Tomography/Computed Tomography (PET/CT) allows noninvasive whole-body (WB) identification of ER distribution and is usually performed as a single static image 60 min after radiotracer injection. Using dynamic whole-body (D-WB) PET imaging, we examine [^18^F]FES kinetics and explore whether Patlak parametric images (${K}_{i}$) are quantitative and improve lesion visibility.

**Results:**

This prospective study included eight patients with metastatic ER-positive BC scanned using a D-WB PET acquisition protocol. The kinetics of [^18^F]FES were best characterized by the irreversible two-tissue compartment model in tumor lesions and in the majority of organ tissues. ${K}_{i}$ values from Patlak parametric images correlated with ${K}_{i}$ values from the full kinetic analysis, r^2^ = 0.77, and with the semiquantitative mean standardized uptake value (SUV_mean_), r^2^ = 0.91. Furthermore, parametric ${K}_{i}$ images had the highest target-to-background ratio (TBR) in 162/164 metastatic lesions and the highest contrast-to-noise ratio (CNR) in 99/164 lesions compared to conventional SUV images. TBR was 2.45 (95% confidence interval (CI): 2.25–2.68) and CNR 1.17 (95% CI: 1.08–1.26) times higher in ${K}_{i}$ images compared to SUV images. These quantitative differences were seen as reduced background activity in the ${K}_{i}$ images.

**Conclusion:**

[^18^F]FES uptake is best described by an irreversible two-tissue compartment model. D-WB [^18^F]FES PET/CT scans can be used for direct reconstruction of parametric ${K}_{i}$ images, with superior lesion visibility and ${K}_{i}$ values comparable to ${K}_{i}$ values found from full kinetic analyses. This may aid correct ER classification and treatment decisions.

*Trial registration* ClinicalTrials.gov: NCT04150731, https://clinicaltrials.gov/study/NCT04150731

**Supplementary Information:**

The online version contains supplementary material available at 10.1186/s13550-024-01080-y.

## Introduction

Breast cancer (BC) is the most common cancer diagnosis among women and the incidence is increasing [1, 2]. Most primary tumors express estrogen receptors (ER) and are considered ER-positive (ER+). Despite advances in treatment, most metastatic BC patients still have poor life expectancy, with a 5-year survival rate of 34–46% for ER+ disease and 12–40% for ER- disease [3]. Targeting ER by hormonal therapy is one of the pillars of BC treatment [4].

The gold standard for ER assessment is immunohistochemistry (IHC), which is used to predict which patients may benefit from endocrine therapy [5, 6]. However, ER expression can show intra- and intertumoral heterogeneity. The discordance rate in ER from primary tumors to recurrence or metastases has been found to vary from 14 to 48% [7–16]. It is recommended to biopsy accessible BC metastases to confirm the diagnosis and to reassess ER status [4, 17]. Despite this, the prospect of implementing tissue biopsies across all metastatic sites is not clinically feasible, as the procedure is invasive and tumor locations can be challenging to access. Furthermore, a substantial intra- and interobserver variation has been documented in pathology reports [18, 19].

Positron Emission Tomography/Computed Tomography (PET/CT) scans can be used to generate whole-body (WB) images depicting tumor lesions throughout the body. 16α-[^18^F]fluoroestradiol ([^18^F]FES) allows noninvasive identification of functional ER distribution [20–23] and can be used to guide treatment decisions [24]. A standard static [^18^F]FES PET/CT, conducted 60 min after radiotracer injection, allows for the use of standardized uptake values (SUV) [23, 25]. A maximum SUV (SUV_max_) ≥ 1.5 g/mL is one of the currently accepted standards for identifying [^18^F]FES-positive disease and reflects functionally ER+ disease [26, 27]. In invasive lobular BC it is also known to detect more metastases than [^18^F]-Fluorodeoxyglucose ([^18^F]FDG) [28]. Several indications for use of [^18^F]FES PET/CT in patients with BC have been proposed, e.g. initial diagnosis of metastatic disease and progression on endocrine therapy [29, 30].

However, a major limitation of the [^18^F]FES radiotracer is the metabolism, excretion, and thereby high physiological uptake in the liver. This can result in undetectable metastases, cold spots, or heterogeneous uptake corresponding to the location of liver metastases[24, 31]. In a study of 91 patients scanned using [^18^F]FES, no liver lesions or lesions directly adjacent to the liver were detectable because of physiological radiotracer uptake in the liver [15]. Therefore, static [^18^F]FES PET/CT is not the optimal imaging technique to detect liver metastases [24, 26, 29].

**Fig. 1:**
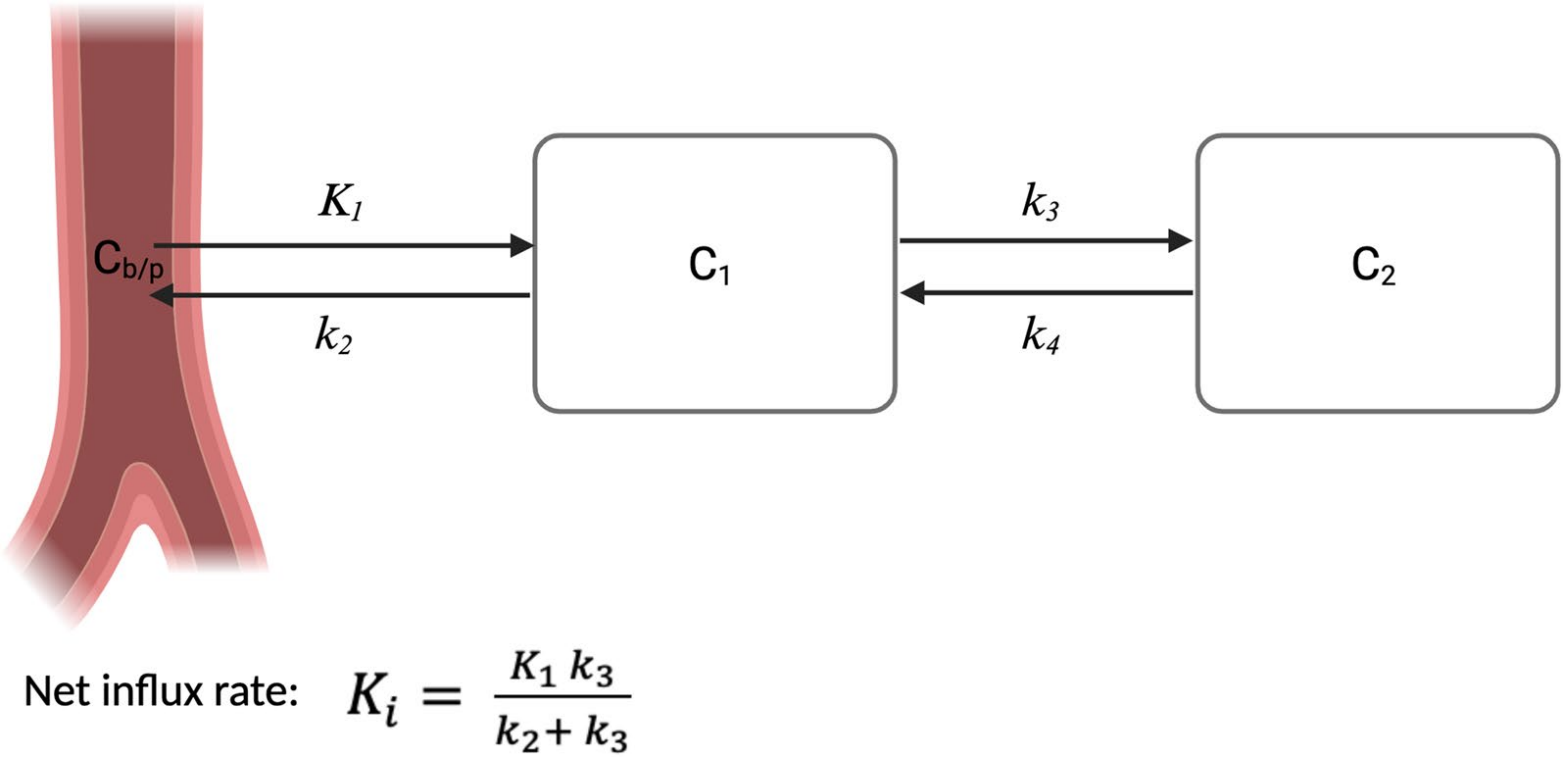
A two-tissue compartment model, in case of irreversible radiotracer uptake *k*_*4*_ = 0. (created with biorender.com)

Recent developments have enabled the generation of dynamic WB (D-WB) PET/CT using multiple WB passes. Based on the Patlak model [32, 33], this technique can, in combination with an irreversible radiotracer uptake, produce additional parametric images, one representing the radiotracer net influx rate (${K}_{i}$), another the distribution volume of free radiotracer in blood and reversible compartments (*V*) [34]. Standard SUV images are a summation of the entire radiotracer signal, whereas ${K}_{i}$ and *V* enable the distinction between bound and free radiotracer. With [^18^F]FDG, it has been shown that the parametric images are superior to SUV images in regard of target-to-background ratios (TBR) and contrast-to-noise ratios (CNR) [35]. However, the uptake pattern (reversible vs. irreversible, Fig. 1) has not been examined for [^18^F]FES. Several studies have conducted dynamic one bed position [^18^F]FES PET/CT [27, 36–38], but none of them examined [^18^F]FES kinetics.

We hypothesized that [^18^F]FES kinetics are best described by a two-tissue compartment model with an irreversible binding to ER, depicted in ${K}_{i}$ images. This study aimed to examine the optimal kinetic model for full quantitative analysis of [^18^F]FES uptake, and, in the case of irreversible radiotracer uptake, examine the tumor visibility in SUV images compared to ${K}_{i}$ images.

## Material and methods

### Ethics and approvals

The protocol received approval from the Danish Medicines Agency (2019083844) and Central Denmark Region Committees on Health Research Ethics (1-10-72-195-19). The study is registered in the European Union Drug Regulating Authorities Clinical Trials Database (EudraCT: 2019-002665-35). The study was monitored by the Good Clinical Practice unit at Aarhus and Aalborg University Hospitals. Written and oral informed consent from all participating individuals were obtained.

### Patients

A total of eight patients were included. Inclusion criteria were (1) metastatic ER+, human epidermal growth factor receptor 2 negative (HER2-) BC (2) at least two liver metastases visualized on CT (3) treatment with aromatase inhibitors or chemotherapy (4) postmenopausal status. Exclusion criteria were (1) one or more ER- metastases (2) treatment with Tamoxifen or Fulvestrant within five weeks of the [^18^F]FES PET/CT scan (3) claustrophobia.

### [^18^F]FES-PET/CT scans

[^18^F]FES was produced in-house at the Aarhus University Hospital radiochemistry facility according to an adopted and modified procedure described by Oh et al. [39], for further detail see Additional file 1.

Patients fasted for a minimum of 6 h before the scan. [^18^F]FES injection was performed as a one-two min infusion followed by injection of saline (NaCl 0.9%). A low dose WB CT (25 Ref mAs, 120 kV, CareDose4D, CarekV, admire level 4) was performed followed by two D-WB scans started directly after the injection of ~200 MBq [^18^F]FES. Patients were scanned on a Siemens Vision 600 PET/CT using the fully automated multiparametric PET acquisition protocol (FlowMotion Multiparametric PET, Siemens Healthineers, Knoxville, USA), starting with a 6 min dynamic scan over the chest followed by 64 min of 16 WB continuous bed motion passes (7 × 2 min WB passes, followed by 9 × 5 min WB passes). The initial scan session ended with an ultra-low dose CT (7mAs). Subsequently, a 10 min intermission was provided to facilitate patient movement and comfort. Following this pause, the patients first had a low dose CT (25 Ref mAs, 120 kV, CareDose4D, CarekV, admire level 4) and the dynamic PET scanning was resumed for a duration of 40 min (8 × 5 min WB passes). The static SUV image was reconstructed using listmode data from 60 to 70 min (reconstruction parameters: TrueX + TOF, 4 iterations, 5 subsets, 440 matrices, 2-mm Gaussian filter and relative scatter correction). The SUV images were normalized to body weight. Parametric images of *K*_*i*_ and *V* were generated using the nested direct Patlak reconstruction method using list-mode data from the 6 last passes, i.e. 40–70 min, and a metabolite-corrected IDIF (reconstruction parameters: TrueX + TOF, 8 iterations, 5 subsets, 30 nested loops, 440 matrices, 2-mm Gaussian filter and relative scatter correction).

Finally, a contrast-enhanced diagnostic CT scan (120 Ref mAs, 120 kV, CareDose4D, admire level 3) was administered, integrated into the standard protocol for patient treatment monitoring. This particular scan served a dual purpose, not only as part of routine observation but also to precisely identify the locations of discernible metastases within the CT images, with a specific emphasis on detecting liver metastases.

### Blood samples

All patients had two venous cannulas placed in a cubital or antebrachial vein, one for tracer injection and one for blood sampling. Venous blood samples were taken before scan start and analyzed for levels of sex hormone binding globulin (SHBG) and estradiol. Additional venous blood samples were taken during the dynamic scan at approximately 2, 5, 10, 20, 40, 60, and 120 min after scan start, comparable to previous studies [40] to estimate the plasma-to-whole-blood fraction and the fraction of unmetabolized [^18^F]FES in plasma. Activity concentrations in plasma and whole blood were measured in a Hidex AMG gamma counter and used to determine the plasma-to-whole-blood fraction. Radio high-performance liquid chromatography (HPLC) (Perkin Elmer series 200 LC pump) was used to measure the fraction of unmetabolized [^18^F]FES in extracts of plasma. For further information on HPLC conditions, see Additional file 1.

### Compartmental modeling

Using the PBAS module in PMOD® 4.0 (PMOD Techmologies Ltd, Zürich, Swtizerland), a volume of interest (VOI) was placed in the descending aorta to extract an image derived input function (IDIF), which was then corrected for metabolites. Time activity curves (TAC) were obtained from the dynamic scans by placing VOIs in normal healthy tissue; liver, gall bladder, lung, heart muscle, muscle, bone, adipose tissue and glandular tissue in the breast, and kidneys. As a consequence of potential patient movement, all time frames were checked manual to ensure the right placement of VOIs. Furthermore, all tumor lesions were contoured using VOIs covering the entire lesion and then restricting it to a 50% iso-contour of SUVmax, this was repeated for all time frames.

Liver volumes were found using AI segmentation on CT, and used to calculate the percentage of injected [^18^F]FES present in the liver over time. The AI segmentation was done using the nn-Unet method based Totalsegmentator tool [41], which is based on the nn-Unet method [42]. Compartmental modeling was conducted using the PKIN module of PMOD®. Data from various tissues, including BC metastases, were fitted to an irreversible and a reversible two-tissue compartment model, see Additional file 1 for elaboration.

Furthermore, kinetics were examined in ER+ BC cells [43–48], see Additional file 1: Fig. S2.

### Lesion visibility

Tumor visibility was examined using quantitative measures TBR and CNR in SUV and ${K}_{i}$ images. For this analysis, lesions with an SUVmax of 1.5 or more were included, background regions were drawn manually in adjacent tissue. TBR and CNR were calculated ﻿as:

$$TBR=\frac{MEAN\left(target \, signal\right)}{MEAN\left(background \, signal\right)}$$

$$CNR=\frac{MEAN\left(target \, signal\right)- MEAN\left(background \, signal\right)}{{\sigma }_{background}}$$

### Statistics

Tumor SUV was presented as median (range). Time series of [^18^F]FES metabolism and TACs are presented as mean ± SEM. The fits of two-tissue reversible and irreversible compartment models were compared by the Akaike information criterion (AIC). A difference in AIC score > 2 was considered a significant difference between models [49]. TBR and CNR values were compared using log-transformation and paired t-tests, results were given as median ratios with 95% CI.

## Results

### Baseline

Median age was 57 years (range 41–74 years). All baseline data are presented in Table S1. Primary tumors were ER+ (range 90%–100%), metastatic lesions were found in lymph nodes (LNs), liver, and bone. ER expression in liver metastases was confirmed by biopsy. Baseline estradiol levels varied among patients but were all within normal range.

Tumor [^18^F]FES uptake varied greatly, with median SUV_mean_ = 3.77 g/mL (range: 1.11–22.56 g/mL) and an SUV_max_ of 5.78 g/mL (range: 1.75–34.43 g/mL). There was no correlation between SUV_mean_ and plasma SHBG, estradiol, or albumin (Additional file 1: Fig. S1).

### Radiotracer blood data

**Fig. 2:**
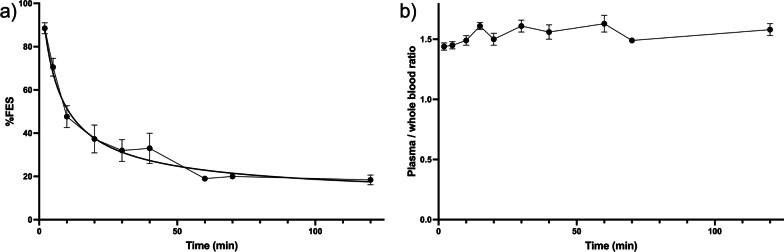
Time course of **a** unmetabolized [^18^F]FES and **b** plasma-to-whole-blood ratio

Mean unmetabolized [^18^F]FES in plasma is shown in Fig. 2a. The initial metabolism was fast with unmetabolized [^18^F]FES dropping from 90% at 2 min to 40% at 20 min. At 120 min ~ 20% remained unmetabolized. The plasma-to-whole-blood ratio showed minimal fluctuation around 1.50 (Fig. 2b).

### Kinetic modeling

**Fig. 3:**
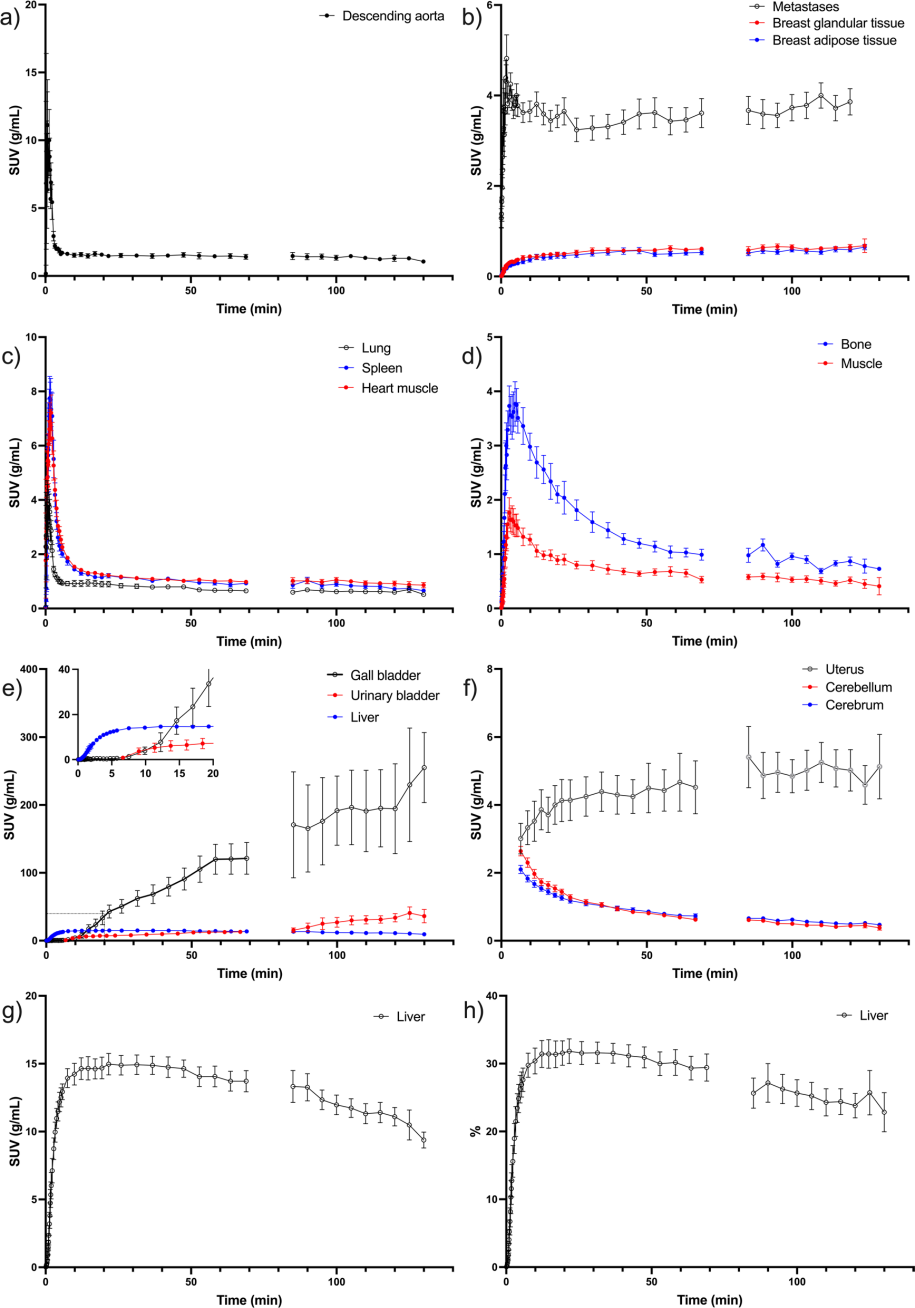
Time activity curves of **a**–**g**: SUVmean and **h** percentage of injected dose in selected tissues

In Fig. 3, tissue TACs are visualized. Glandular and adipose tissue of the breast showed identical patterns with continuous low activity around SUV_mean_ 0,6. The TAC in metastases was characterized by a rapid increase, which 1 min post-injection reached SUV_mean_ ~4.5 where it remained. Activity in the heart muscle, lung, and spleen followed the blood curve. Muscle and bone had an activity peak at 5 min, then a decrease leveling out at SUV_mean_ 1. Gall bladder activity was observed after 5–10 min, it increased intensely throughout the remaining scan time. The same pattern was evident for the urinary bladder, although the increase was less pronounced. Activity in the uterus stayed constant after the initial 30 min at SUV_mean_ 4–5, while brain activity decreased from 6 min and onwards. The liver demonstrated a rapid initial activity increase, maintained an elevated level until 50 min after injection, after which a gradual decrease commenced. From 10–50 min, 30% of injected [^18^F]FES was found in the liver, then the percentage slowly decreased.

**Fig. 4:**
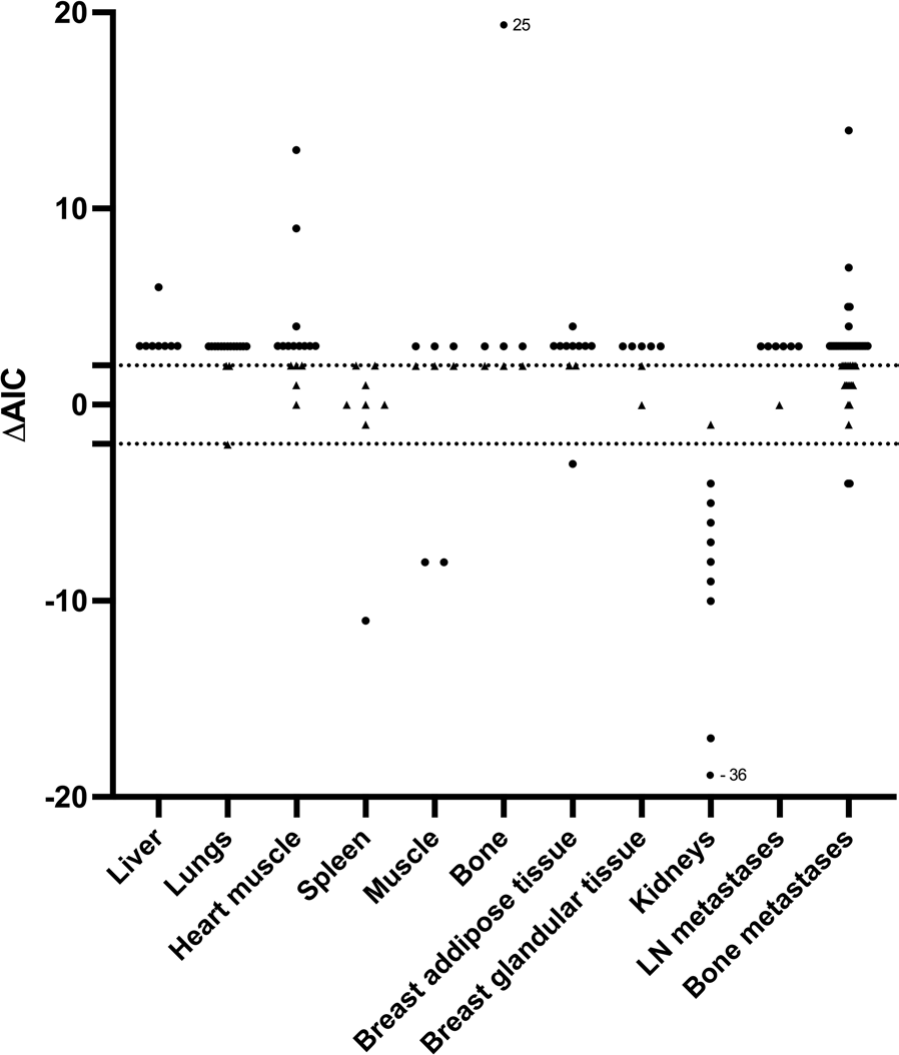
ΔAIC for organs and metastatic lesions. ΔAIC = AIC (reversible two-tissue compartment model)—AIC (irreversible two-tissue compartment model). As the model with the lowest AIC is the best fit, a positive ΔAIC reflects that the irreversible two-tissue compartment model is the best fit, while a negative ΔAIC reflects that the reversible two-tissue compartment model is the best fit. Black triangles have ΔAIC ≤ 2 while black dots have ΔAIC > 2 (ΔAIC > 2 is considered a significant difference between models)

The kinetics of [^18^F]FES exhibited optimal characterization by the irreversible two-tissue compartment model in the majority of organ tissues (Fig. 4). ΔAIC decisively supported an irreversible kinetic model in liver, lungs, heart muscle, bone, glandular, and fatty tissue of the breast. In the spleen and muscle tissue, no distinct inclination towards either model was evident, while kidney activity was best described by the reversible model. Radiotracer uptake in metastatic tumor lesions in LN and bone was best characterized by an irreversible model.

**Fig. 5:**
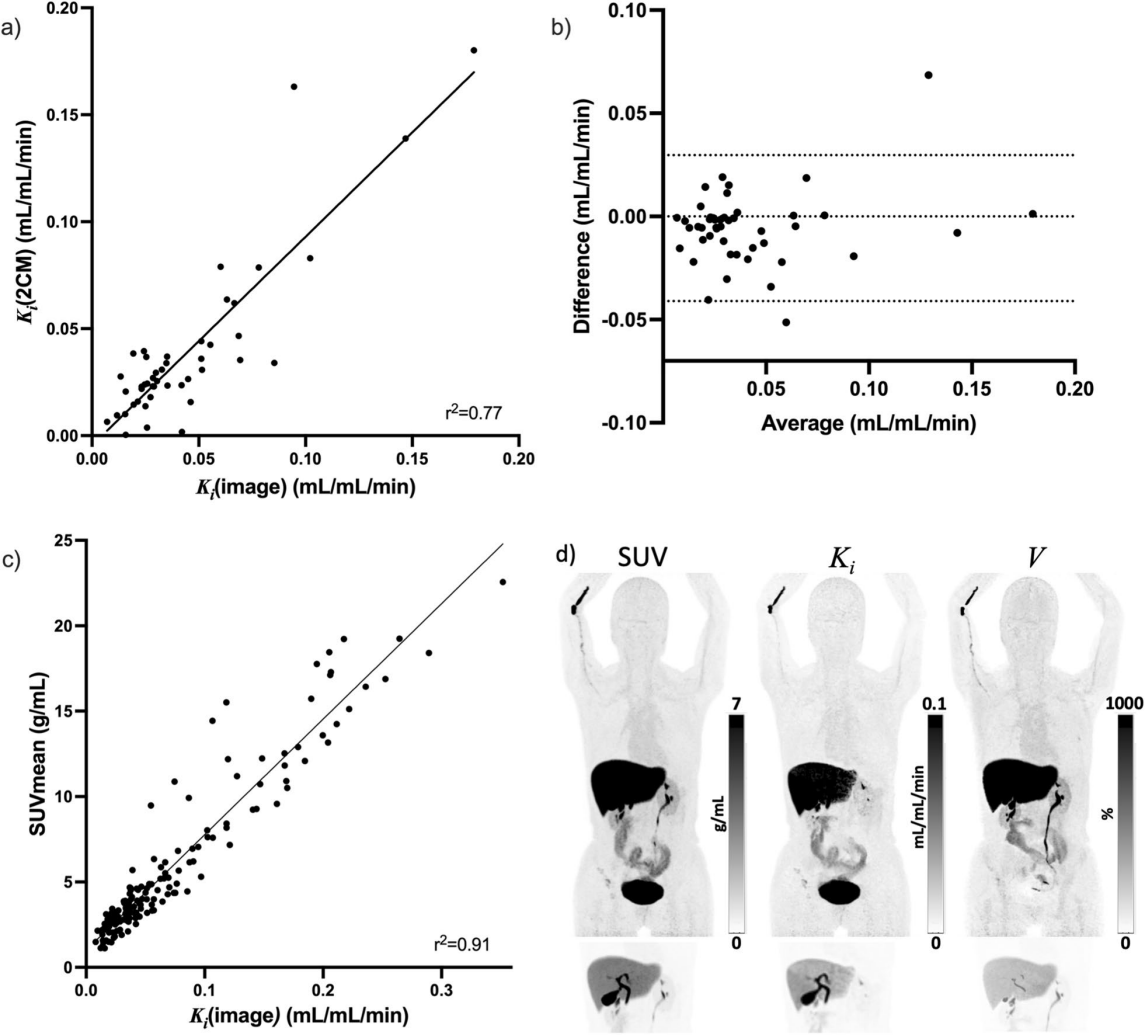
**a** Correlation between ${K}_{i}$ from the Patlak reconstruction image, ${K}_{i}$(image), and ${K}_{i}$ from the full kinetic analyses using the irreversible two-tissue compartment model, **b** Bland–Altman analysis of difference vs. average, **c** Correlation between ${K}_{i}\left({\text{image}}\right)$ vs. SUVmean, and **d** image reconstructions from D-WB [^18^F]FES PET/CT, the bottom line visualizes liver and biliary passage with 3.5 times lower intensity

In metastatic lesions with full dynamic scan data (0–70 min), a correlation between ${K}_{i}$ from the direct reconstruction from PET raw data (${K}_{i}$(image)) and ${K}_{i}$ from indirect image-based full kinetic analyses (${K}_{i}$(2CM)) was found, r^2^ = 0.77 (Fig. 5). Including all metastatic lesions, the correlation between the quantitative parameter ${K}_{i}$(image) and semiquantitative SUV_mean_ was excellent, r^2^ = 0.91.

As described in Additional file 1, [^18^F]FES kinetics were examined in ER-expressing BC cells. This analysis supported our in vivo results and demonstrated that the irreversible binding played a significant role throughout the latter part of the elimination phase. See Additional file 1 for full description.

### Quantitative lesion visibility

**Fig. 6:**
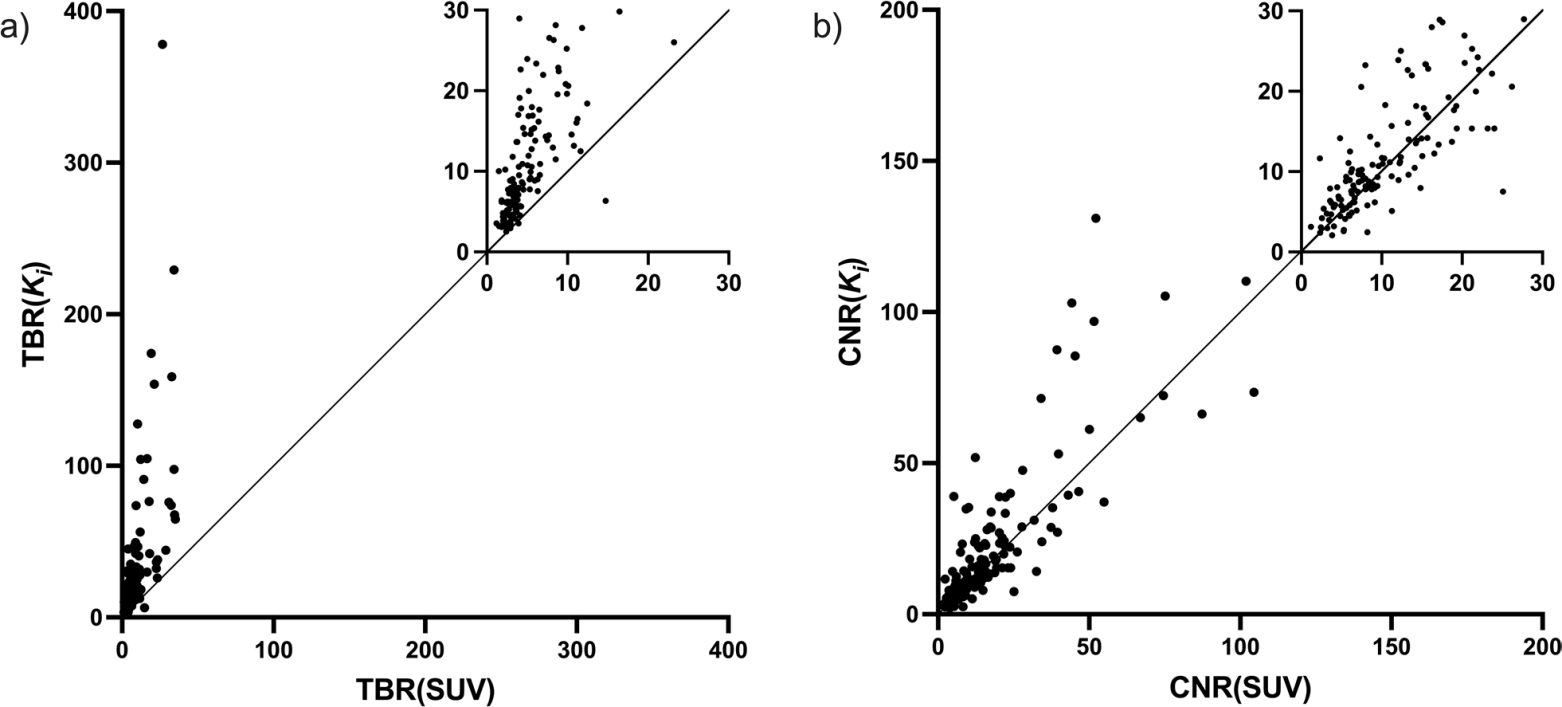
Correlation between **a** target-to-background and **b** contrast-to-noise ratios in SUV and ${K}_{i}$ images

As the irreversible two-tissue compartment model was the overall best fit, lesion visibility was examined by TBR and CNR in standard SUV images (TBR(SUV) and CNR(SUV)) and compared to values from ${K}_{i}$ images (TBR(${K}_{i}$) and CNR(${K}_{i}$)), results are depicted in Fig. 6. TBR(${K}_{i}$) was highest in 162 of 164 (99%) metastatic lesions. TBR(${K}_{i}$) was 2.45 (95% CI: 2.25–2.68) times higher than TBR(SUV). CNR(${K}_{i}$) was highest in 99/164 (60%) metastases, and CNR(${K}_{i}$) was 1.17 (95% CI: 1.08–1.26) times higher than CNR(SUV).

**Fig. 7:**
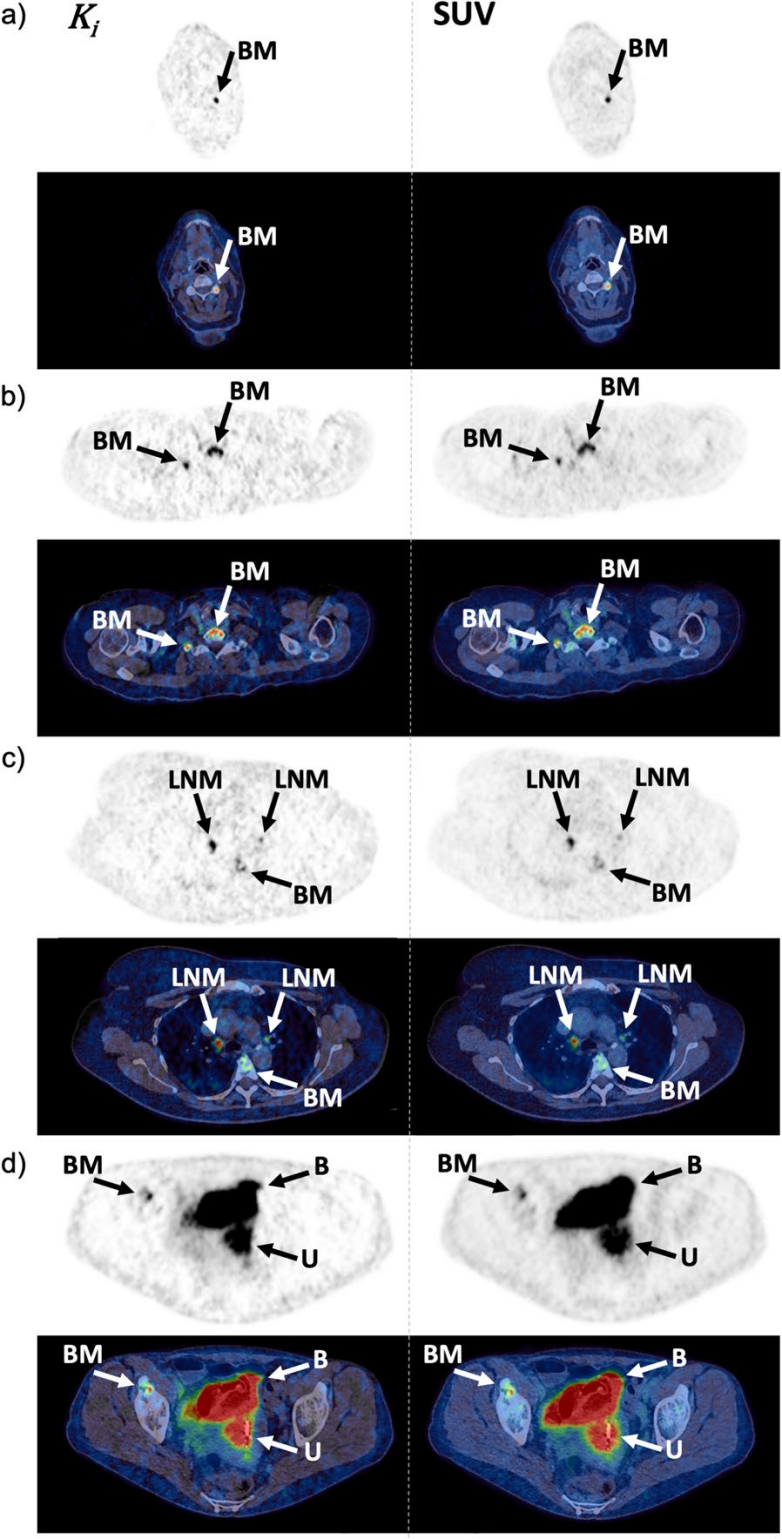
Examples of the better TBR and CNR for bone metastases (BM) and LN metastases (LNM) in ${K}_{i}$ images compared to SUV images. Metastases were located in **a** C5, **b** C7, **c** T5 and mediastinal lymph nodes, and **d** pelvic bone, where the bladder (B) and uterus (U) were also present

**Fig. 8:**
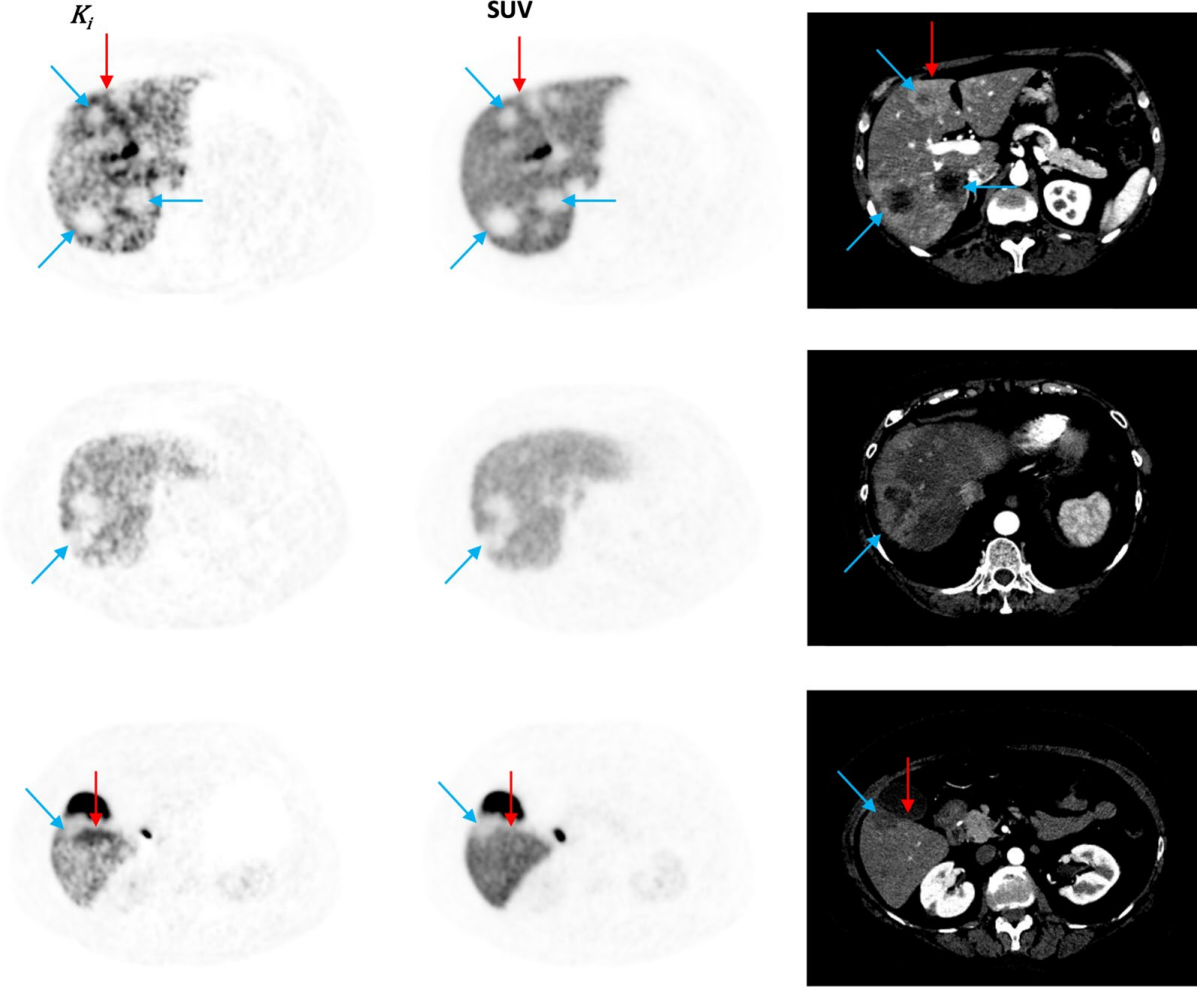
Different appearances of liver metastases in ${K}_{i}$ images, SUV images, and CE-CT. Blue arrows represent lesions with necrotic centers appearing as cold spots on [^18^F]FES PET/CT. Red arrows represent lesions visible on ${K}_{i}$ images

This quantitative difference was also evident in the visual assessment where background activity was reduced in ${K}_{i}$ images (Fig. 7). Analyses of [^18^F]FES kinetics in liver metastases were not possible due to spill-in from liver tissue. Background activity in liver tissue was reduced in ${K}_{i}$ images, however this did not aid in the detection of liver metastases. Several different appearances of liver metastases were noticed (Fig. 8). Lesions with a diameter > 15 mm and a necrotic center on contrast-enhanced CT (CE-CT), appeared as cold spots in both SUV and ${K}_{i}$ images. A few lesions had increased values on ${K}_{i}$ images, most were located in the vicinity of large necrotic lesions. Small lesions (< 10 mm) were, in general, undetectable in both SUV and ${K}_{i}$ images.

## Discussion

In this study, kinetic analyses of [^18^F]FES uptake was found to be best described by an irreversible two-tissue compartment model. Overall, TBR was highest in ${K}_{i}$ images, which have the potential to improve the detection of ER+ lesions. Furthermore, as ${K}_{i}$(image) compared well with ${K}_{i}$(2CM), simple acquisitions of [^18^F]FES kinetic parameters are possible using a metabolite-corrected IDIF.

Improvement of tumor lesion visibility in parametric PET images has been demonstrated for various cancer forms using various PET radiotracers [35, 50]. In these studies, direct reconstruction of parametric images from PET raw data was conducted using the IDIF extracted automatically from the aorta. However, an aorta IDIF cannot be directly used for D-WB [^18^F]FES PET/CT scans, due to the radiotracer metabolism. A direct transfer of automated IDIF extraction would result in an IDIF with an overestimated area under the curve (AUC). In the Patlak plot, an overestimation of the AUC of the plasma input function will lead to an underestimation of ${K}_{i}$. To manage this, analyses of [^18^F]FES metabolism were conducted and were similar to what has been found by others [40, 51, 52]. The plasma/whole-blood ratio was constant throughout the scan, which is also similar to previous reports [51]. This metabolite-corrected input function was used for the direct reconstruction of parametric images.

In the present study no correlation between plasma SHBG and tumor SUV, or between estradiol and SUV, was observed despite that previous studies have suggested that SUV measurements should be corrected for plasma SHBG and fractional [^18^F]FES binding to SHBG [53, 54]. Peterson et al. recommended correcting SUV measurements for plasma SHBG, however, there was great variation in their reported SUV measurements for all SHBG levels, with no clear pattern [54]. As such, further studies should be conducted before the implementation of any adjustment to SUV measures is possible.

TACs of numerous normal tissues and BC lesions were reported. TACs have not previously been described for heart muscle and spleen, nor bone and LN metastases. Previous reported TACs for breast tissue, muscle, bone, lung, and liver were compatible with the ones found in this study [51]. TACs in the cerebrum and cerebellum were almost identical, and in agreement with preliminary results from Ghanzafari et al. (under review). Furthermore, the SUV value at 60 min fits well with a case description reporting a SUVmean of 1.16 in a standard [^18^F]FES PET/CT scan[55]. In the uterus, the activity stayed constant at SUVmean ~4.5 from 20 min post injection and onwards. There is a discrepancy between our results and previously reported uptake in the uterus [56]. However, present results are in agreement with Beauregard et al. who examined the uterus activity of 4-Fluoro-11β-methoxy-16α-^18^F-fluoroestradiol [57], another estradiol analog for PET imaging [58]. [^18^F]FES metabolites are known to be excreted into bile [56]. In accordance with this, we found an intense activity increase in the gall bladder. Activity in the urinary bladder increased simultaneously with the gall bladder, indicating that some of [^18^F]FES or its metabolites are excreted without entering the enterohepatic circulation.

The activity in most metastases did not exceed the general activity in the liver. Assuming that the liver metastases are compatible with other BC lesions, this can explain why it is so challenging to visualize liver metastases on [^18^F]FES PET/CT [23, 24]. Moreover, it is noteworthy that the direct reconstruction of parametric images currently lacks the capability for data-driven motion compensation. In the future, the potential integration of respiratory gating into the reconstruction of ${K}_{i}$ images could lead to enhanced image quality, particularly in organs like the liver. We found that liver metastases with a diameter > 15 mm that appeared with necrotic centers on CE-CT were seen as cold spots on both SUV and ${K}_{i}$ images. It was possible to locate some lesions on ${K}_{i}$ images. However, despite the multiparametric scan protocol, it still proved challenging to visualize liver metastases, and this remains a major limitation of the [^18^F]FES scan. All patients fasted for 6 h prior to the scan, as this is standard procedure, in our institution, before a CE-CT due to the risk of an anaphylaxis reaction. Fasting has been suggested to reduce bowel accumulation due to bile excretion [29], whether this can also slow down the metabolism of [^18^F]FES is unknown. Regardless, reliable identification of tumor lesions in the liver might be impossible using this radiotracer. Other tracers, like [18F]FDG and [68Ga]FAPI, might help in detecting liver metastases but do not offer insights into ER expression [59]. Therefore, to meet this goal, there is an ongoing need to develop new tracers that specifically target ER and exhibit minimal binding or metabolism in the liver.

Kinetic analysis showed that [^18^F]FES uptake was best described by the irreversible two-tissue compartment model. This was further supported by kinetic analysis of [^18^F]FES excretion in BC cell culture. ${K}_{i}$ values from the multiparametric reconstructions correlated well with ${K}_{i}$ from the full two-tissue compartment analyses, furthermore, TBR and CNR favored ${K}_{i}$ images. These quantitative measurements were reflected in the images, where background activity was diminished, in line with previous reports [35, 50]. In the kidneys, the kinetics of [^18^F]FES was best described by a reversible model, probably due to a low, if any, ER expression [60] and excretion of radiolabeled metabolites.

This study has some limitations. Our data suggested that lesion detectability was better in ${K}_{i}$ images. However, as BC metastases were identified from the SUV image using a limit of SUVmax 1.5, we did not examine whether the utilization of parametric images would lead to the identification of additional lesions. The extensive nature of the scan protocol prolonged the inclusion period as few patients, who fulfilled the inclusion criterion of multiple liver metastases, were able to complete a two-hour continuous scan. As this was predictable, only eight patients were included in the study. The full dynamic scan only covered one bed position, constraining the full kinetic analysis to tissues and lesions in the chest and upper abdomen. This obstacle will be eliminated with the implementation of long-axial field-of-view scanners, which will both increase the number of tissues available for analysis and decrease image noise. Another limitation is the lack of concurrent biopsies to compare [^18^F]FES uptake with IHC in all lesions. However, the study's rationale includes addressing the impracticality of conducting multiple biopsies in the case of metastatic BC.

## Conclusions

[^18^F]FES uptake is best described using an irreversible two-tissue compartment model. D-WB [^18^F]FES PET/CT scans can be used to generate automated ${K}_{i}$ images, with superior lesion visibility and ${K}_{i}$ values comparable to ${K}_{i}$ values from full kinetic analyses. Further studies are needed to assess if the superior lesion visibility can lead to the identification of additional tumor lesions, in which case it may aid correct ER classification and treatment decisions. However, the reliable identification of tumor lesions in the liver may be unachievable with this radiotracer, despite the application of the irreversible two-tissue compartment model. Therefore, efforts should be directed towards developing new, improved radiotracers that have a strong affinity for the ER.

### Supplementary Information

**Additional file 1**. Supplemental material.

## Abbreviations

[^18^F]FES: 16α-[^18^F]fluoroestradiol
AIC: Akaike information criterion
BC: Breast cancer
CNR: Contrast-to-noise ratio
D-WB: Dynamic whole-body
ER: Estrogen receptor
HER2: Human epidermal growth factor receptor 2
HPLC: High-performance liquid chromatography
IDIF: Image derived input function
IHC: Immunohistochemistry
PET/CT: Positron emission tomography / computed tomography
SHBG: Sex hormone binding globulin
SUV: Standardized uptake value
SUV_max_: Maximum standardized uptake value
TAC: Time activity curves
TBR: Target-to-background ratio
VOI: Volume of interest
WB: Whole-body

## Declarations

### Availability of data and materials

The data that support the findings of this study is available from the corresponding author upon reasonable request.
