# Supplementary material for: Dynamic whole-body [18F]FES PET/CT increases lesion visibility in patients with metastatic breast cancer
Source: EJNMMI Res. 2024 Mar 4;14:24. doi: 10.1186/s13550-024-01080-y (PMC10912074; doi:10.1186/s13550-024-01080-y)
Supplement: Supplementary file 1 — Additional file 1. Supplemental material. [file 13550_2024_1080_MOESM1_ESM.docx]

# Dynamic whole-body [^18^F]FES PET/CT increases lesion visibility in patients with metastatic breast cancer

Mette A. Pedersen^1,2,3^, Ole L. Munk^1,4^, André H. Dias^1^, Johanne H. Steffensen^5^, Anders L. Møller^5^, Anna Lyhne Johnsson^6^, Kim Vang^1^, Dirk Bender^1,4^, Steen Jakobsen^1^, Morten Busk^7,8^, Lars C. Gormsen^1,4^, Trine Tramm^4,9^, Signe Borgquist^4,5^, Mikkel H. Vendelbo^1,2,3^

^1^ Department of Nuclear Medicine & PET Centre, Aarhus University Hospital, Aarhus, Denmark

^2^ Department of Biomedicine, Aarhus University, Aarhus, Denmark

^3^ Steno Diabetes Center Aarhus, Aarhus University Hospital, Aarhus, Denmark

^4^ Department of Clinical Medicine, Aarhus University, Aarhus, Denmark

^5^ Department of Oncology, Aarhus University Hospital, Aarhus, Denmark

^6^ Department of Radiology, Aarhus University Hospital, Aarhus, Denmark

^7^ Department of Experimental Clinical Oncology, Aarhus University Hospital, Aarhus, Denmark

^8^ Danish Centre for Particle Therapy, Aarhus University Hospital, Aarhus, Denmark

^9^ Department of Pathology, Aarhus University Hospital, Aarhus, Denmark

Corresponding author: Mikkel H. Vendelbo (mikkvend@rm.dk)

SUPPLEMENTAL MATERIAL

# Methods

## [^18^F]FES production

[^18^F]FES was produced with a validated process according to an adopted and modified procedure as described by Oh et al. [1]. on commercially available reagent kits and cassettes, ABX advanced biochemical compounds GmbH, product numbers PEFS-0024H and PEFS-0024R, Radeberg, Germany on a GE Healthcare™ Tracerlab Mx synthesis platform. Briefly, after cyclotron irradiation [^18^F]-fluoride is trapped and separated from the irradiated O‑18 water on Waters QMA Sep-Pak™ cartridge, eluted from the QMA Sep-Pak cartridge using 500 μL of tetrabutylammonium bicarbonate (TBA·HCO_3_ solution, 0.075 M). After drying, [^18^F]-fluoride is reacted with 1.5 mg of 3‑methoxymethyl-16β,17β-epiestriol-*O*-cyclic sulfone (MMSE) in 1.5 mL acetonitrile at 130^o^C for 8 min. Protective groups are removed within a consecutive acid hydrolysis. Product purification and isolation is performed on a combination of a Waters Oasis WAX™, Waters Oasis C18™ and Waters Oasis HLB™ solid phase extraction cartridges. After isolation and purification ethanol content is reduced to an acceptable content by distillation and the product is transferred through a Waters Alumina N cartridge™ and a 0.22 µm sterile filter in the final product vial already containing 15 mL sterile water. After QC the product is released for application.

## [^18^F]FES-PET/CT scans

Patients fasted for a minimum of 6 hours before the scan. [^18^F]FES injection was performed as a one-two min infusion followed by injection of saline (NaCl 0,9%). A low dose WB CT (25 Ref mAs, 120 kV, CareDose4D, CarekV, admire level 4) was performed followed by two D-WB scans started directly after the injection of ~200 MBq [^18^F]FES. Patients were scanned on a Siemens Vision 600 PET/CT using the fully automated multiparametric PET acquisition protocol (FlowMotion Multiparametric PET, Siemens Healthineers, Knoxville, USA), starting with a 6 min dynamic scan over the chest followed by 64 min of 16 WB continuous bed motion passes (7 x 2 min WB passes, followed by 9 x 5 min WB passes). The initial scan session ended with an ultra-low dose CT (7mAs). Subsequently, a 10 min intermission was provided to facilitate patient movement and comfort. Following this pause, the patients first had a low dose CT (25 Ref mAs, 120 kV, CareDose4D, CarekV, admire level 4) and the dynamic PET scanning was resumed for a duration of 40 min (8 x 5 min WB passes). The static SUV image was reconstructed using listmode data from 60 to 70 min (reconstruction parameters: TrueX+TOF, 4 iterations, 5 subsets, 440 matrices, 2-mm Gaussian filter and relative scatter correction). The SUV images were normalized to body weight. Parametric images of *K_i_* and *V* were generated using the nested direct Patlak reconstruction method using list-mode data from the 6 last passes, i.e. 40–70 min, and the automatically generated IDIF (reconstruction parameters: TrueX+TOF, 8 iterations, 5 subsets, 30 nested loops, 440 matrices, 2-mm Gaussian filter and relative scatter correction).

Finally, a contrast-enhanced diagnostic CT scan (120 Ref mAs, 120 kV, CareDose4D, admire level 3) was administered, integrated into the standard protocol for patient treatment monitoring. This particular scan served a dual purpose, not only as part of routine observation but also to precisely identify the locations of discernible metastases within the CT images, with a specific emphasis on detecting liver metastases.

## Blood samples

To measure the fraction of unmetabolized [^18^F]FES in extracts of plasma, radio high-performance liquid chromatography (HPLC) (Perkin Elmer series 200 LC pump) was used. HPLC conditions were LUNA C18(2) 5-μm 100 column (Phenomenex, 250 x 10 mm) with an eluent consisting of 60% aqueous 70 mmol/L Na2HPO4 and 40% acetonitrile delivered at 8 mL/min. Detection consisted of serial UV detection (λ = 220 nm) and radio detection. All samples were further fractionated and collected in 30 second intervals and measured for radioactivity in a Hidex AMG gamma counter. The retention time of [^18^F]FES was 6-8 min as determined with an authentic sample.

## Compartmental modeling and image reconstruction

Compartmental modeling was conducted with data from various tissues, including BC metastases, and were fitted to an irreversible and a reversible two-tissue compartment model. In these models, [^18^F]FES in the blood plasma is reversible absorbed to the intracellular space in different tissues, this reversible absorption is described by the rate constants $K_{1}$ and $k_{2}$. [^18^F]FES then binds to its receptor in the nucleus and is involved in gene transcription activation [2]. This receptor binding is described by the rate constants $k_{3}$ and $k_{4}$, where $k_{4}$ is negligible in the case of an irreversible binding.

SUV images represent a semiquantitative measure of tissue radiotracer uptake, with values defined as tracer concentration corrected for injected dose, patient body weight, and radioactive decay. Parametric $K_{i}$ images, based on the Patlak reconstruction model, were reconstructed from PET raw data using the metabolite corrected input function. They represent the net influx constant $K_{i}$ in the case of an irreversible uptake. $K_{i}$ images were generated with data from 40 to 70 min.

## Cell experiments

MCF7 breast cancer cells [3,4] (expressing ER) were cultured in high-glucose (450 mg/l glucose) Dulbecco's Modified Eagle Medium (D-MEM) supplemented with 10% fetal bovine serum, pyruvate (1 mM), non-essential amino acids and 2% HEPES buffer. For experiments, 1x10^6^cells resuspended in 2 ml medium were seeded in a defined area, in a tilted 100mm Petri dish and placed in an incubator. The next day, following firm adherence of cells, dishes were flooded with an additional 8 ml of medium and returned to the incubator. Cells were used for experiments 4 days after seeding. Prior to measurements, dishes were scraped outside the intended cell seeding area, to avoid the confounding influence of cells outside the measurement area. Dishes were next supplemented with 5 ml of fresh pre-equilibrated (incubator) medium and placed in a LigandTracer apparatus (model: white), which was placed in a cell incubator to ensure accurate temperature and CO2 control. After a short baseline measurement, ~0.77 MBq/mL of [^18^F]FES was added to the cells and cellular accumulation of [^18^F]FES was recorded. After an appropriate cell tracer-loading period, the Petri dish was thoroughly rinsed and supplemented with 5 ml fresh tracer-free medium, and tracer excretion was monitored for a period of 70 min. The subsequent decay- and background-corrected time-activity curves representing the tracer's uptake and excretion were methodically plotted and analyzed. This quantitative analysis was executed utilizing the Ligand Tracer White device (Ridgeview Instruments, Uppsala, Sweden) [5–7].

Each experiment consisted of an uptake phase and an elimination phase, with the last data point in the uptake phase set to 100%. The elimination phase data included time points and corresponding percentages of maximum. Two missing data points from one of the three experiments were excluded from the analysis because of a machine malfunction.

The elimination kinetics was fitted to a single- (1) and bi-exponential (2) elimination model:

$f\left( t \right)=Ae^{\left( -kt \right)}+C$ (1)

$f\left( t \right)=A_{\text{1}}e^{\left( -k_{\text{1}}t \right)}+A_{\text{2}}e^{\left( -k_{\text{2}}t \right)}+C$ (2)

*A* = amplitude of the exponential component, *k* = elimination constant, *C* = constant component and *t* = time in min. The model was fitted to data using nonlinear least-squares optimization in the curve fit function in Scientific Python (Python Software Foundation) [8]. Initial guesses for the parameters were derived from the data, and mean parameters across the three experiments were calculated.

The single-exponential and bi-exponential decay models were compared based on the coefficient of determination (R^2^), with higher R^2^ values indicating a better fit.

Analysis of contribution of the constant C (irreversible binding) relative to the reversible part at time t was calculated using the following ratio:

$$Contribution=\frac{C}{C+ A_{\text{1}}e^{\left( -k_{\text{1}}t \right)}+A_{\text{2}}e^{\left( -k_{\text{2}}t \right)}}$$

# Results

## Kinetic modeling in breast cancer cells

The elimination phase was best described by the bi-exponential model: $f\left( t \right)=45.01e^{\left( -0,30t \right)}+4,18e^{\left( -0.034t \right)}+8.93$, R^2^=0.9994 (Figure S3). We examined cell data from 40 to 70 min post-injection, corresponding to the time span used to generate human $K_{i}$ images. This analysis demonstrated that the irreversible binding, played a significant role throughout the latter part of the elimination phase. During this specific timeframe, the average contribution of irreversible binding ranged between 88.74% (95% CI: 84.11-93.37) and 94.91% (95% CI: 91.60-98.22).

# Tables

| Table S1: Baseline characteristics | |
| --- | --- |
| **Pathology** |  |
| Primary tumor  Ductal/lobular/missing (n)  ER% (range)  HER2 (range)  Ki-67 (range)  Grade (range)  Liver metastases  ER% (range)  HER2 (range)  Ki-67 (range) | 6/1/1  90-100%  1+ - 2+  <5 – 70  I – II  100%  1+ - 2+  70 |
| **Current treatment** |  |
| Aromatase inhibitors n (%)  Cytostatic drugs n (%) | 6 (75%)  2 (25%) |
| **Blood samples** |  |
| Albumin (range)  SHBG (range)  Estradiol (range) | 36 – 41 g/l  23 – 107 nmol/l  <15 – 63 pmol/l |

# Figures


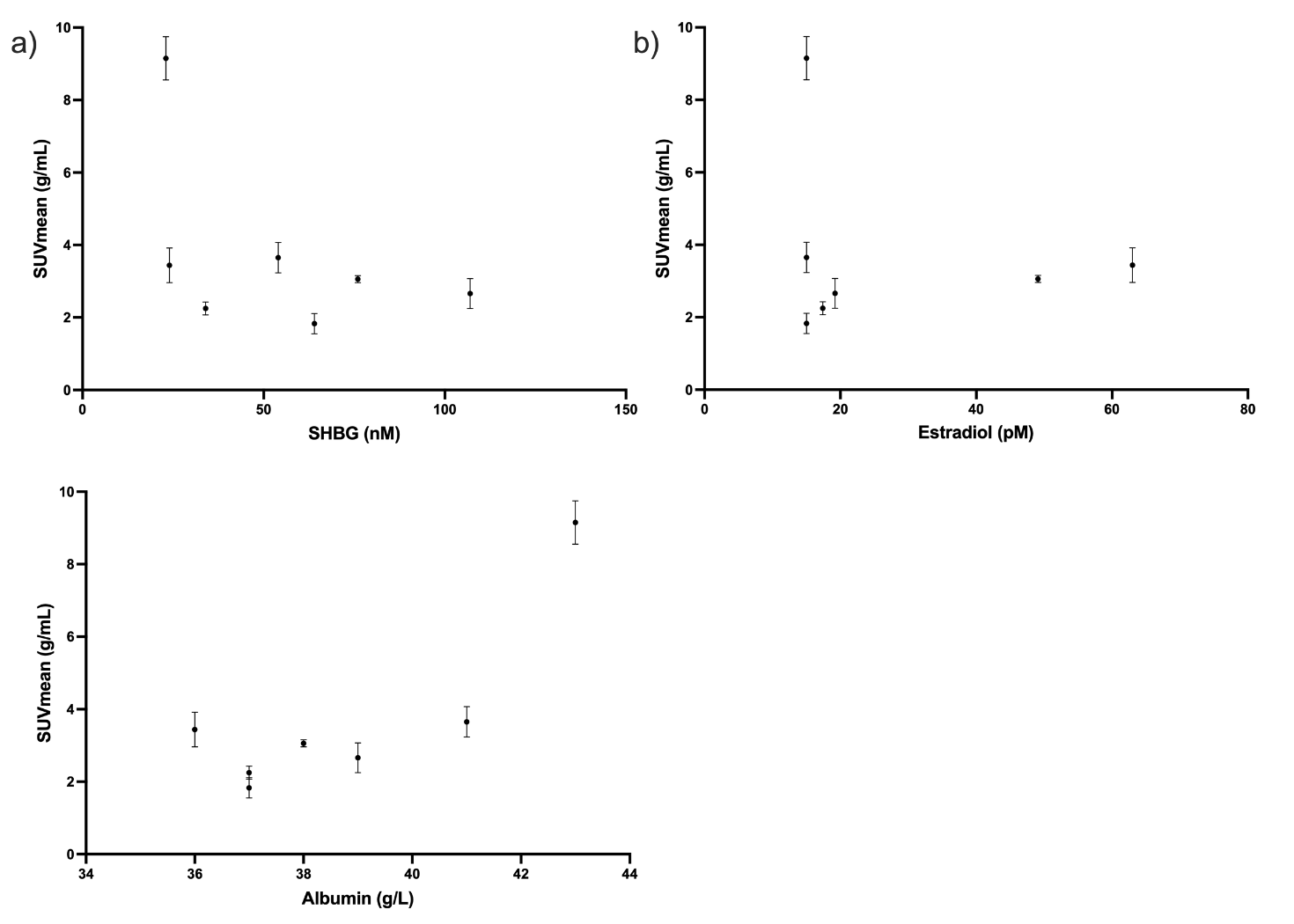


Supplemental Figure S1: Correlation between the mean SUVmean for all lesions in each patient and SHBG, estradiol, estrone, and albumin


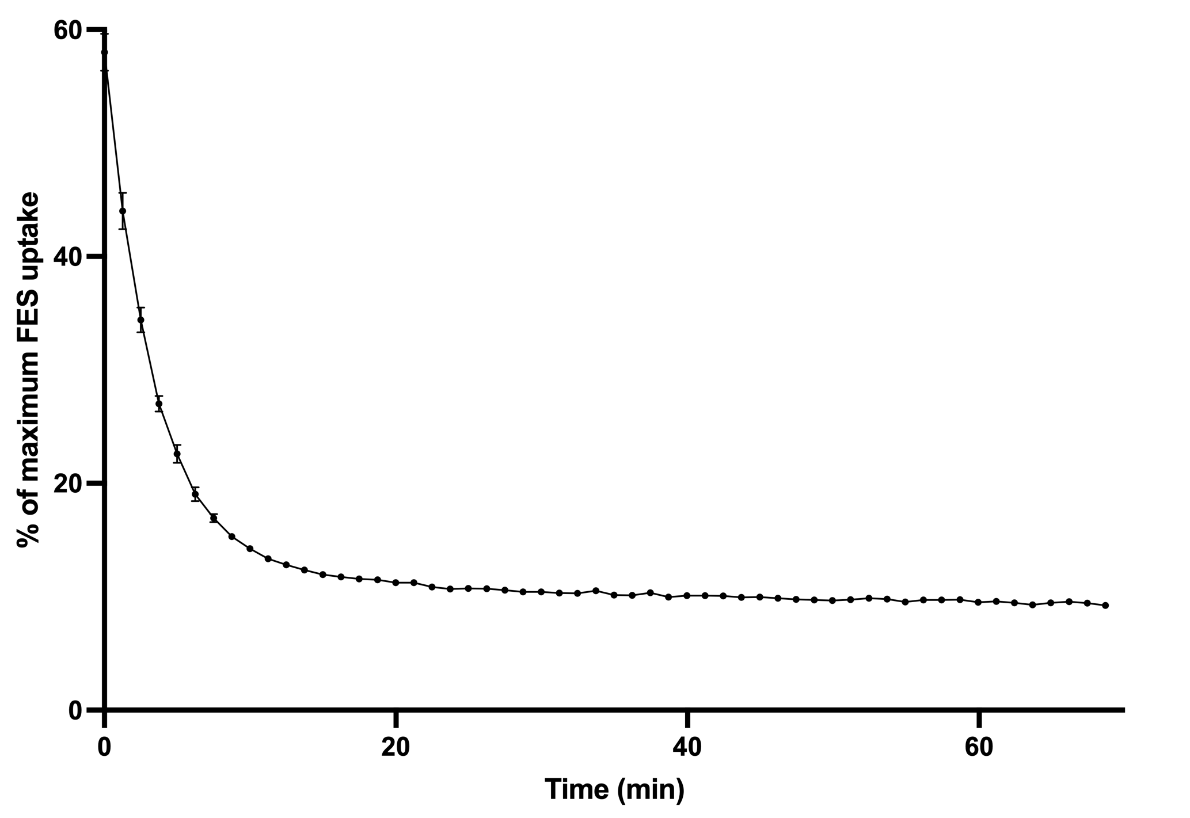


Supplemental Figure S2: Full curve pattern of [^18^F]FES elimination in MCF7 breast cancer cells.

References

1. Oh SJ, Chi DY, Mosdzianowski C, Kil HS, Ryu JS, Moon DH. The automatic production of 16α-[18F]fluoroestradiol using a conventional [18F]FDG module with a disposable cassette system. Appl Radiat Isot. 2007;65:676–81.

2. Fuentes N, Silveyra P. Estrogen receptor signaling mechanisms. Adv Protein Chem Struct Biol. 2019;116:135–70.

3. Comşa Ş, Cîmpean AM, Raica M. The story of MCF-7 breast cancer cell line: 40 Years of experience in research. Anticancer Res. 2015;35:3147–54.

4. Soule HD, Vazquez J, Long A, Albert S, Brennan M. A human cell line from a pleural effusion derived from a breast carcinoma1,2. J Natl Cancer Inst. 1973;51:1409–16.

5. Björke H, Andersson K. Automated, high-resolution cellular retention and uptake studies in vitro. Appl Radiat Isot. 2006;64:901–5.

6. Björke H, Andersson K. Measuring the affinity of a radioligand with its receptor using a rotating cell dish with in situ reference area. Appl Radiat Isot. 2006;64:32–7.

7. Busk M, Sinning S, Alstrup AKO, Munk OL, Vendelbo MH. Nuclear Medicine Preclinical Research: The Role of Cell Cultures. Semin Nucl Med. 2023;53:558–69.

8. Virtanen P, Gommers R, Oliphant TE, Haberland M, Reddy T, Cournapeau D, et al. SciPy 1.0: fundamental algorithms for scientific computing in Python. Nat Methods. 2020;17:261–72.
